# “You Get Reminded You’re a Sick Person”: Personal Data Tracking and Patients With Multiple Chronic Conditions

**DOI:** 10.2196/jmir.4209

**Published:** 2015-08-19

**Authors:** Jessica S Ancker, Holly O Witteman, Baria Hafeez, Thierry Provencher, Mary Van de Graaf, Esther Wei

**Affiliations:** ^1^ Weill Cornell Medical College Department of Healthcare Policy and Research Division of Health Informatics New York, NY United States; ^2^ Laval University Faculty of Medicine Department of Family and Emergency Medicine Quebec City, QC Canada; ^3^ Laval University Faculty of Medicine Office of Education and Continuing Professional Development Quebec City, QC Canada; ^4^ Research Centre of the CHU de Quebec Quebec, QC Canada; ^5^ Laval University Quebec City, QC Canada; ^6^ Weill Cornell Medical College Department of Medicine New York, NY United States

**Keywords:** medical informatics, consumer health information, health knowledge, attitudes, practices, self-care, chronic disease

## Abstract

**Background:**

Consumer health information technologies (HIT) that encourage self-tracking, such as diet and fitness tracking apps and disease journals, are attracting widespread interest among technology-oriented consumers (such as “quantified self” advocates), entrepreneurs, and the health care industry. Such electronic technologies could potentially benefit the growing population of patients with multiple chronic conditions (MCC). However, MCC is predominantly a condition of the elderly and disproportionately affects the less affluent, so it also seems possible that the barriers to use of consumer HIT would be particularly severe for this patient population.

**Objective:**

Our aim was to explore the perspectives of individuals with MCC using a semistructured interview study. Our research questions were (1) How do individuals with MCC track their own health and medical data? and (2) How do patients and providers perceive and use patient-tracked data?

**Methods:**

We used semistructured interviews with patients with multiple chronic diseases and providers with experience caring for such patients, as well as participation in a diabetes education group to triangulate emerging themes. Data were analyzed using grounded theory and thematic analysis. Recruitment and analysis took place iteratively until thematic saturation was reached.

**Results:**

Interviews were conducted with 22 patients and 7 health care providers. The patients had an average of 3.5 chronic conditions, including type 2 diabetes, heart disease, chronic pain, and depression, and had regular relationships with an average of 5 providers. Four major themes arose from the interviews: (1) tracking this data feels like work for many patients, (2) personal medical data for individuals with chronic conditions are not simply objective facts, but instead provoke strong positive and negative emotions, value judgments, and diverse interpretations, (3) patients track for different purposes, ranging from sense-making to self-management to reporting to the doctor, and (4) patients often notice that physicians trust technologically measured data such as lab reports over patients’ self-tracked data.

**Conclusions:**

Developers of consumer health information technologies for data tracking (such as diet and exercise apps or blood glucose logs) often assume patients have unlimited enthusiasm for tracking their own health data via technology. However, our findings potentially explain relatively low adoption of consumer HIT, as they suggest that patients with multiple chronic illnesses consider it work to track their own data, that the data can be emotionally charged, and that they may perceive that providers do not welcome it. Similar themes have been found in some individual chronic diseases but appeared more complex because patients often encountered “illness work” connected to multiple diseases simultaneously and frequently faced additional challenges from aging or difficult comorbidities such as chronic pain, depression, and anxiety. We suggest that to make a public health impact, consumer HIT developers should engage creatively with these pragmatic and emotional issues to reach an audience that is broader than technologically sophisticated early adopters. Novel technologies are likely to be successful only if they clearly reduce patient inconvenience and burden, helping them to accomplish their “illness work” more efficiently and effectively.

## Introduction

### Background

Consumer health information technology (HIT) is exploding in popularity, attracting the attention of technology-oriented consumers, patients, caregivers, and entrepreneurs. Technologies such as disease management apps and “quantified self” tools [1-3] offer the potential to help patients track personal data, learn about their health, and manage chronic care needs [4-7]. Consumer HIT appears poised to help inform, motivate, and engage patients, all of which are known to improve management skills and health outcomes [5-8].

However, it is not yet known whether such technologies will diffuse broadly beyond tech-savvy early adopters such as “quantified self” advocates, and whether the technologies would produce benefits for people with complex medical conditions. To date, the measured impact of consumer HIT is still limited. Computerized interventions for diabetes self-management have shown only limited efficacy [9,10]. In practice, effects have generally been limited as a result of low adoption and usage. One in 5 smartphone users has downloaded a health app [11], yet most apps are abandoned after a few uses [12]. Studies of the effectiveness of apps and websites to promote health outcomes (such as a recent study of a phone app to assist in weight loss [13] or a self-management Web community for diabetes [10]) frequently find that participants stop using the technology after a short period of time. Having a chronic condition increases the chances that a patient will use certain forms of consumer HIT on average [2,11]. But this increased likelihood is often offset by other sociodemographic factors that decrease the likelihood of using technology. Of particular concern from a public health standpoint, the use of consumer HIT remains lowest among the groups that might be most likely to benefit from additional forms of low-cost disease management support: people who are elderly, less educated, or less affluent [2,11,14]. These disparities in uptake, as well as the low rate of sustained use among adopters, suggest mismatches between current consumer HIT and the goals, desires, or capabilities of many patients [15,16].

A population with particularly complex and ongoing health needs is the 90 million Americans who have multiple chronic conditions (MCC) [17]. Although any combination of chronic conditions qualifies as MCC, the most common combinations are diabetes plus hypertension, heart disease plus hypertension, and cancer plus hypertension [18]. Patients with MCC experience the challenges associated with living with chronic disease and also typically consult more different doctors and coordinate more different therapeutic regimens than those with single diseases [17]. Each additional chronic condition places the individual at higher risk of adverse drug events, out-of-pocket expenses, impaired functional status, hospitalization, and mortality [17]. It is estimated that two-thirds of health care spending is focused on patients with MCC [17].

These patients are in need of improved strategies and technologies to support health and medical care, creating a number of opportunities that could potentially be filled with health IT, yet the barriers to technology adoption might be particularly problematic for these patients as well. MCC disproportionately affects the elderly and the less affluent. The prevalence of MCC rises sharply with age, affecting 34% of those aged 45-64 and 62% of those age 65 and over [19]. Furthermore, the prevalence of MCC is highest among the lowest income brackets, affecting nearly 51% of seniors who live at or below the federal poverty level but only 39% of seniors living at four times the poverty level [18].

As an initial step to exploring the perspectives of individuals with multiple chronic conditions, with the goal of understanding potential applications of consumer HIT and barriers to its use, we conducted a semistructured interview study. This paper focuses on tracking or keeping diaries of personal data, a task that we will refer to as “personal health information tracking”. We focused on personal health information tracking because (1) it has been recommended for a variety of chronic conditions, and (2) it is a task potentially supported by consumer health IT. Self-monitoring tasks that have been promoted under different circumstances include blood glucose self-monitoring for certain patients with type 1 and type 2 diabetes [20,21], measuring blood pressure in hypertension and heart disease [22], keeping diet logs or food diaries for weight loss or digestive diseases [23], and self-monitoring medication adherence and side effects [24]. Patients also often receive the recommendation that they should check and be able to report certain laboratory values, such as CD4 count in human immunodeficiency virus (HIV) or hemoglobin A1c in diabetes. We therefore considered personal health information tracking to be a task that was likely to be encountered by patients with MCC, but we did not a priori assume a position on whether patients should self-track or whether it was likely to benefit them. Rather, our research questions were (1) How do individuals with MCC perform medical data tracking? and (2) How do patients and providers perceive and use patient-tracked data? We asked the questions broadly to encompass any sort of tool the patients were currently using, including electronic technologies, paper, or memory.

### Theoretical Framework: Illness Work and Personal Health Information Management

This project was conducted from a human factors perspective influenced by the sociology of illness. This perspective recognizes that patients’ management of their health comprises a wide variety of different activities both inside and outside the medical encounter: taking medicines, refilling prescriptions, buying and cooking food, exercising or doing physical therapy, researching health issues, coping with medical crises, finding doctors and dentists, organizing and traveling to medical appointments, and keeping records. As these are all effortful, directed activities to attain goals, they may be conceptualized as work [25-27].

Corbin and Strauss identified “illness work” as activities directly involved with managing an illness, such as following medication regimens and using technologies such as glucose meters or sleep apnea machines [25,26]. Yet even in illness, “everyday life work” of shopping, paying bills, nurturing relationships, and managing a household continues [25,26]. “Articulation work” is the planning, coordinating, and managing that allows people to complete all their other work [25,26].

Those components of illness and articulation work that involve acquiring and managing information can be called personal health information management [27-30]. A growing body of research on personal health information management has identified tasks including tracking health events, obtaining information, and organizing information [27]; creating personal histories, making decisions, planning, and structuring activities (eg, creating medication reminders) [28]; and transferring personal data and records to the physician [31]. In the current project, we focus on the subset of personal health information management involved in monitoring and logging personal data (such as symptoms or laboratory values), sometimes called personal health information tracking [32].

Much of the recent work in personal health information tracking and management has focused on generally healthy individuals and families [27,28,31,32], on patients with cancer [33-36], or (in support of information technology design) on computer-literate participants [31].

In this project, we sought to apply the insights from this previous work while exploring the perspectives of an economically diverse sample of patients with MCC in more depth. In order to develop or adapt technologies for these patients, it is essential to understand practices and perspectives of the potential users and the attributes of the tasks they seek to perform, as well as the social and physical environments in which they will be performing these tasks [15]. Poor fit between individuals, tasks, and technologies is likely to be one of the reasons that self-tracking technologies have not yet spread widely within populations with multiple diseases.

## Methods

### Participants

For individual interviews, we recruited purposive samples of adult English-speaking patients with MCC, and of medical providers with experience providing care for patients with MCC. We adopted the Department of Health and Human Services definition of chronic conditions as conditions that last a year or more and that either require ongoing medical attention or limit activities of daily living [17]. Patient participants were recruited from outpatient clinics in internal medicine and endocrinology and from the patient information library, using both promotional flyers and individual referrals from physicians and nurse practitioners. One researcher (JSA) also attended six 90-minute sessions of a diabetes education support group as a means of triangulating emerging themes. We chose the diabetes education group because many of the study participants had type 2 diabetes.

### Settings

Weill Cornell Physicians is a multispecialty academic medical practice in Manhattan, with a mix of privately insured, Medicaid, and Medicare patients. New York-Presbyterian Hospital is the largest academic medical center in Manhattan. The Institute for Family Health is a federally qualified health center with 18 sites in and around New York City, providing safety net primary care to patients regardless of insurance status.

### Interview Methods

The researchers developed a semistructured interview instrument centered on three topics: personal health information tracking, personal health information management, and searching for health-related information. The current manuscript focuses on the first of these. The first author conducted interviews in person, using offices and conference rooms convenient to the clinics where patients were recruited. Interviews were audio recorded and professionally transcribed. The interviewer also took field notes, collected samples of artifacts and documents for patients such as educational brochures, and photographed other artifacts or documents such as log sheets used to record glucose values.

### Analysis Methods

No existing theoretical framework appeared to be appropriate to these data, and therefore we applied methods to develop meaning inductively from the data. Although this family of approaches is sometimes known in the sociology literature as development of grounded theory [37], we adopt the newer term “inductive thematic analysis” to reflect the fact that our end product is a series of interrelated themes rather than a fully formed theory [38]. Qualitative analysis was conducted collaboratively by our multidisciplinary team, which included individuals with training in journalism, public health, informatics, psychology, human factors, nursing, and diabetes education. Two of the researchers (HOW and EW) also brought personal experience of long-standing chronic disease. The preliminary version of the codebook was developed by 2 of the researchers in reading the first three transcripts and was iteratively refined over the coding process. Each transcript and photograph was reviewed by at least 2 team members (the first author and one other team member), who independently coded the transcript and then met to reach consensus on it.

We followed a staged and iterative approach, first identifying preliminary codes through repeated reading and review of the data, then identifying relationships between codes and groupings of codes, and finally identifying and refining larger underlying themes. Over the analysis, 47 open codes were developed. These were linked into 6 broad groups: (1) resources, skills, and factors patients need for disease management, (2) the health care system and its components, (3) thinking, feeling, and experiencing disease and health, (4) medical data and medical records, (5) evaluative judgments, and (6) attributions of responsibility. In the final stage, the themes presented in the results section were developed.

To improve internal validity, we conducted member checking [39] in two ways. First, several of the emergent groups and themes were presented to new informants during interviews for their feedback. Second, the resulting themes were presented at a meeting of the diabetes education group, whose members validated the themes while also providing additional feedback and nuanced interpretation.

Analysis and recruitment were conducted simultaneously until saturation was achieved (ie, no new concepts were arising from new interviews) [40].

This study was approved by the Institutional Review Boards of Weill Cornell Medical College and the Institute for Family Health. All participants gave written informed consent. Members of the diabetes education group provided oral consent.

## Results

### Participants

Interviews were conducted with 22 patients and 7 health care providers. An additional 3 patient interviews were excluded from analysis because the interviewees did not have multiple chronic conditions.

The included patients reported having an average of 3.5 chronic conditions (SD 1.5). The most common conditions mentioned were type 2 diabetes, hypertension, heart disease, chronic pain, and depression. Other conditions included asthma, HIV, hepatitis C, thyroid disorders, rheumatoid arthritis, glaucoma, cataracts, and sleep apnea. Two individuals were in follow-up after cancer treatment. Conditions reported by only one patient each included type 1 diabetes, fibromyalgia, post-polio syndrome, sarcoidosis, Sjogren syndrome, and cirrhosis. Many described themselves as overweight but none as obese. In addition to their chronic conditions, patients also discussed a wide variety of recently experienced urgent conditions, including diverticulitis, flu, appendicitis, bee stings, and physical injuries. Participants sometimes mentioned taking drugs that implied other chronic conditions that they did not explicitly list: examples included antidepressants, blood pressure medications, lipid-lowering medications, drugs for prostatic hyperplasia, and anticoagulants. Many of the patients with type 2 diabetes were taking insulin one or more times a day, as was the individual with type 1 diabetes.

Half of patients were men and half were women; a third (n=7) were black. Ages ranged from 37-89 (mean 64.1; median 66). About two-thirds (n=15) were not currently married. Just over a third (n=8) used English as a second language. One third (n=7) were covered by Medicare (US public insurance for those over age 65); one third (n=7) by Medicaid (US public insurance for those with low income); and the remainder (n=8) by commercial insurance.

Multiple chronic conditions placed heavy and sometimes competing demands on patients. For example, one patient with diabetes recognized that his morning toast caused increases in his blood glucose, but on balance had decided not to stop eating toast because his morning medications for other conditions had to be taken with food. Several patients with diabetes or heart disease recognized that exercise might help but were prevented because of chronic pain or disability from injury. Patients taking anticoagulants encountered challenges when scheduling surgery for other conditions.

The diabetes education group was attended by an average of 5 patients each session (range 4-9). Most patient education group attendees had type 2 diabetes but a minority had type 1 diabetes or prediabetes.

The health care providers were 2 nurse practitioners, 2 internists, 2 family medicine physicians, and an emergency medicine physician.

Major themes pertaining to personal health information tracking are summarized in Table 1 and presented in detail in the results section.

**Table 1:** Major themes in personal health information tracking.

Themes	Summary	Representative quotes
1. Personal data can carry strong emotional and moral implications	Data are not merely objective facts but prompt strong positive and negative emotions as well as value judgments.	“You get reminded you’re a sick person” and “I’m not a good patient”.
2. Multiple purposes and uses for personal data	Patients use data for a variety of purposes, ranging from active self-management to making sense of their condition to reporting to the doctor.	“I’ll [check] it if I’m feeling lightheaded”.
3. (Un)reliability of personally tracked data	Patients often notice that physicians do not trust their self-tracked data.	“[The doctors] looked at [my logs] very superficially…they seem to rely on your A1c numbers”.
4. Tracking feels like work	Tracking is time-consuming and sometimes emotionally draining.	“It’s too cumbersome for me”.

### Overview

Most patients paid attention to laboratory findings provided by their doctors, and a few kept records of selected values. For example, a woman with anemia created a table to track her blood test results over time, 2 patients with HIV kept records of their CD4 count values over time, and many patients checked on their cholesterol regularly.

However, fewer than half regularly tracked data by self-testing or recording daily activities. The most common example of self-tracking was patients with diabetes monitoring their blood glucose. Among the 16 patients with type 1 or type 2 diabetes, 11 mentioned self-monitoring blood glucose in some fashion (some were fairly regular, some checked values occasionally, and some said they used to monitor regularly but had stopped). Other examples of tracking mentioned by one or more participants included recording weight or blood pressure (n=7), tracking daily medication administration (n=3), keeping food diaries (n=2, in one case to investigate suspected lactose intolerance), collecting laboratory reports to manually compare trends over time (n=4), and recording potential side effects with a new medication (n=2). This sort of tracking was conducted on paper or electronically on a spreadsheet, or in one case on a paper calendar. All the patients interviewed who monitored blood glucose used monitors that tracked data electronically. In addition, some kept handwritten blood glucose logs. The numbers in parentheses above (n=) are provided for perspective, but these data were collected through open-ended interview questions rather than closed-ended survey methods, so the interviews may not have captured every instance of tracking.

Many of the patients older than 65 and most Medicaid patients did not use computers regularly or at all, and many did not have smartphones.

### Theme 1: Personal Data Can Carry Strong Emotional and Moral Implications

#### Overview

Indicators such as blood glucose, weight, and lab values were not discussed as value-free facts but instead carried strong emotional and evaluative connotations. People recognized tracking as work, judged themselves as “good” or “bad” for their data and their diligence in collecting it, and noted that data should be considered within the patient’s personal context.

#### Negative Aspects of Illness

Medical data often reminded patients of the negative aspects of their illness. An individual who did not monitor her blood glucose regularly said her values were “depressing”, and another said they made her “scared”. Discussing tracking sometimes raised feelings of anger or injustice not only about the tracking but also about having chronic disease. “I hate to be focused on my health in every friggin’ second of the day...I don’t want to live like that every day”. A patient with HIV, hypertension, and other chronic illnesses said he avoided looking at his regular test results: “I don’t ask about no numbers. If anything is messing up, then [my doctor] tells me”. The physical experience could also be unpleasant. “Poking my finger, that was irritating to me,” said one person who had abandoned blood glucose self-monitoring. “I’m tired of sticking myself,” another said. Some patients with diabetes said they were frustrated to see their blood glucose values occasionally spike without a clear reason, undermining their confidence that they understood and could manage their disease.

#### The Moral Valence of Medical Data

Patients and providers frequently described the data with highly judgmental language, including terms suggesting moral transgression. For example, one explained a high blood glucose value because “I cheated and I had some McDonald’s”. Conversely, patients could feel extremely happy and proud when their values were good. Several of the health care providers said it was better to use nonjudgmental language such as “high/low” or “target/nontarget” because patients “get discouraged because they think they’re being graded or judged”. Yet in the interviews, many providers used more evaluative language such as “good/bad” and “better/worse”. A patient who had altered his diet and was able to lower his doses of hypertension and hyperlipidemia drugs said he felt satisfied when his doctors said, “Okay, we’re happy with you”.

#### The Moral Valence of Tracking

There was also a “good/bad patient” aspect to tracking itself. People with diabetes frequently called themselves a “bad patient” or “not a good patient” when they did not monitor blood glucose. One participant explained the fact that she did not track any of her health indicators (including diet and exercise) by calling herself “lazy”. Although providers most often expressed frustration about lack of monitoring, some occasionally perceived monitoring as excessive. Patients who tracked data very diligently (eg, detailed exercise logs, which clinicians saw as having little clinical relevance) were sometimes referred to as “obsessive and compulsive” or “fastidious”.

#### My Interpretation of My Data

Although in some cases patients and physicians were in close agreement about what data values were “good” or “bad”, other patients preferred to interpret their results in light of their own unique histories or symptoms. For example, several patients with diabetes said that they aimed for a blood glucose level or hemoglobin A1c that was appropriate “for me”. In some cases, these were values that made them feel well, or values that were high enough to minimize the risk of hypoglycemia. In other cases, patients wanted their personal history to be taken into account in interpreting data. For example, a person with a history of obesity took pride in the number of dress sizes she had gone down, rather than aiming for a particular target weight. One provider told an anecdote about a patient who had brought her hemoglobin A1c from 13% to below 8% with diet and medication. When urged to continue lowering it, the patient said, “I don’t want to be a poster child for perfect diabetes”. The doctor recalled saying, “Actually, you’re right. This is good for you...I should’ve been jumping up and down because that’s really great”.

### Theme 2: Multiple Purposes and Uses for Personal Data

#### Overview

Not all patients closely monitored their own data values. Patients who did track their own data through either self-monitoring or laboratory testing described a variety of purposes, which depended on aspects of their disease and on their own experience of their disease. They might use their tracked data for real-time decision making, for medium-term self-assessment, or for making sense of various elements of data, such as physical symptoms.

#### Tracking for Action

Some experienced patients with diabetes monitored blood glucose multiple times per day as “working data” [30] that they would use immediately to adjust their diet or their medication. For example, one woman described a highly effective routine of using thrice-daily glucose monitoring to adjust sliding-scale medication doses and diet. She had used these techniques to reduce her hemoglobin A1c level to 6.1% for nearly a year. Most health care providers perceived this active, real-time use of data for self-management as important for patients who were struggling to manage conditions in which data values were highly sensitive to behavior (such as a younger patient with new-onset diabetes), but less important for others (such as older patients with stable disease).

#### Tracking for Goal-Checking

A second approach was to use data periodically to assess progress toward a goal. Patients with this approach referred to the data for a holistic assessment of how “well” they were doing, but not necessarily for active, hour-to-hour self-management. This was also often the approach used by patients who were monitoring indicators that they themselves could not measure, such as cholesterol, blood count values in anemia, HIV viral load levels, and CD4 counts.

#### Tracking for Sense-Making

A different approach was to examine data values as part of trying to make sense of the disease. Several patients with diabetes who did not regularly monitor described checking glucose when they felt symptoms they suspected indicated hypoglycemia: “I’ll do it if I’m feeling lightheaded”. Another said he did it when he felt a “hunch”. This approach was sometimes encouraged by physicians for patients who seemed unlikely to monitor regularly: “Usually I tell them that if they’re not feeling well, check their blood sugar”. One patient with HIV asked his doctor for explanations whenever his lab values changed. “I saw this is different [from] last 2-3 months ago, and now something is wrong. And he explained to me if it’s something wrong or not [important]”. During visits, health care providers frequently explicitly linked lab values to patient behavior to encourage them to develop a more biomedical concept of the disease. For example, one provider used a patient’s headache as a teaching example to discuss the role of salt in her diet. Some also saw it as a useful short-term exercise for patients seeking an understanding of behavioral triggers for conditions such as asthma, irritable bowel syndrome, or migraine headaches. However, some patients described frustration (or even abandoning tracking altogether) after failing to see connections between their data values and their behavior.

#### Tracking for the Doctor

A few patients appeared to perceive self-monitoring as something done not for their own use but partly or largely to create records for the doctor. A few seemed confused that doctors rarely reviewed their logs. “They don’t monitor that part of it, I don’t know why”.

### Theme 3: (Un)reliability of Personally Tracked Data

Providers often perceived patient-recorded data as unreliable. The lack of confidence was attributed to perceived lack of diligence, moral valence of the data (with patients unwilling to “admit” undesirable numbers), and fear of consequences. The most striking example, told by a provider, was a woman who faked her daughter’s blood glucose log to persuade the doctor to delay starting insulin therapy.

Providers sometimes described lab data as more trustworthy than data from self-tracking. “The hemoglobin A1c don’t lie [sic], so you can tell me whatever you want, but it’s going to tell me the truth of what’s going on in your body”. Another said: “For the most part a lot of this information I don’t really [need] because I can check the A1c and know what it’s like”. Current diabetes treatment guidelines recommend attention to self-monitored blood glucose for extreme values and trends, in addition to hemoglobin A1c as an indicator of overall control [20,21].

These perceptions on the part of providers were evident to many of the patients. “I remember when I used to go to the diabetes center up there with [a doctor] and she looked at it very superficially too, and they seem to rely on your A1c numbers,” said a patient who had abandoned logging his daily glucose values. Providers also sometimes perceived automated recording devices as more reliable than patient-recorded information, which was also noticed by some of the patients: “[My doctor] is like, ‘Please bring me the machine’”. One provider told an anecdote about a patient with a dangerous blood pressure increase; the patient’s spouse used a monitor to print out the previous week’s blood pressure readings, which were low enough to persuade the doctors to rule out their initial suspicion of “medication noncompliance”.

In only one case, a highly engaged patient said that her provider preferred reviewing her blood glucose logs rather than the glucose monitor because the log made it easier to link the readings to meals. “It was a lot of confusion with the doctor because I was just bringing the machine. So now [with the notebook] they know that first one, two, three is breakfast, lunch, and dinner”.

### Theme 4: Tracking as Work

Patients said that tracking was effortful and time-consuming, sometimes explicitly describing it as work. A patient with diabetes said it was a waste of time to write down her values: “I’m not going to sit down and write a paper for the month to keep track of it”. One woman noted that she kept medical information about her multiple conditions, as well as her multiple health care providers, in her office rather than her home. Data tracking sometime was felt to conflict with the work of everyday living forcing trade-offs when patients did not have sufficient time or emotional resources. A diabetes patient who had given up self-monitoring of blood glucose said, “It’s too cumbersome for me”. A patient with heart disease who kept a diet log gave it up after it became “overwhelming”.

## Discussion

### Principal Findings

Developers of consumer health information technologies for data tracking (such as diet and exercise apps or blood glucose logs) often assume patients have unlimited enthusiasm for tracking their own health data via technology, that these data are objective facts with unambiguous interpretations and applications, and that health care providers welcome such data in their assessment of a patient’s health status. Potential users are believed to be “willing to assume a more participatory role in the management of their health, to learn how to use new tools, and to commit themselves to doing so constantly” [31].

By contrast, the concept of data tracking as patient work was strongly supported by our interviews with patients with multiple chronic conditions. Furthermore, personal medical data did not appear to be objective facts, interpreted in the same way by patients and their providers. The data provoke strong negative and positive emotional reactions, sometimes overwhelming ones that prevent people from wanting to track or access their data. These data can also make individuals feel judged by their health care providers or even by themselves. Patients may resist their physician’s interpretation of their data values as “one-size-fits-all” and may prefer to weight their own personal history and disease experience. Physicians often trust technologically measured data more than manual self-tracked data; their preference is apparent to patients and may inadvertently be sending patients mixed messages about the value of their data tracking efforts.

Our study also suggests that patients who do keep track of their data require it for different purposes. Some patients examine their data periodically for a holistic check on their own progress toward goals, and others use their data for real-time decisions about their behavior. Yet another group of individuals inspect and interpret this data as part of the process of developing an understanding of their disease.

Finally, we encountered many elderly and low-income patients who had limited experience with and access to electronic technologies. As our sample was fairly representative of the demographics of those with MCC (with a mean age of 64 and about one third covered by Medicaid), it is plausible that this reflects the experience of broader MCC populations.

These findings support the proposal that existing self-tracking technologies such as mobile phone apps may not provide a good fit to the needs and abilities of individuals with MCC and the tasks they are seeking to perform with them [15].

### Comparison With Prior Work

Our work contributes to a growing body of research in personal health information management and personal health information tracking—research that has already identified a range of tasks frequently performed by patients, ranging from tracking health data to managing medical records to creating personal reminder systems [27-29,31,32]. However, much of the previous work in this field has focused on generally healthy individuals and families [27,28,31,32], on patients with cancer [33-36], or (in support of HIT design) on computer-literate participants [31].

The current project identifies different perspectives brought by an economically diverse group of patients with multiple chronic diseases. Our participants each had several chronic diseases, including diabetes, HIV, heart disease, depression, and many others, and about one third were covered by Medicaid. Their perspectives were in many cases different from what has been found in previous work with healthy families. For example, while healthy consumers in Canada rejected the idea that health information management was “work” [32], our patients with MCC frequently described managing data as time-consuming and tiring. There are several potential explanations for this contrast. First, keeping track of even a single chronic disease is likely to be more challenging than keeping track of preventive care or minor medical events among largely healthy individuals. Second, individuals with multiple chronic conditions are likely to have “illness work” connected to each of the diseases (our patients had an average of 3.5 chronic conditions). Third, MCC is disproportionately a condition of the elderly as well as the less affluent, meaning that an MCC patient may be conducting “illness work” while simultaneously facing challenges related to aging and poverty. Finally, the multiple chronic conditions included physically and emotionally challenging comorbidities such as depression, anxiety, and chronic pain—conditions that themselves might make it more difficult to conduct any “illness work”. This workload burden may have been particularly evident as many of our patients were unmarried and had primary responsibility for their own personal health information. By contrast, previous research with families often shows that one family member takes primary responsibility for the information needs of the household [27,28,31,32]. Such a division of labor within the family context might offer several advantages, including the ability for the information manager to specialize and develop expertise in information management, and might also alleviate the workload burden on more ill members of the household.

Our findings support previous work in the field of technology development for elderly patients or others who do not use electronic technologies regularly. The people we spoke with conducted personal health information management and tracking with a variety of paper and electronic tools, both custom-made and adapted, as has been found by other researchers [27,29,31]. As others have found, we found that older patients and those with Medicaid were frequently unfamiliar with electronic technologies. In addition to lack of access, some have found that elderly patients may find usability barriers discouraging them from adopting new technologies [41]. We additionally found that some adults with experience of chronic disease have already solved their own data management problems to their own satisfaction and did not express much interest in novel technologies. Similarly, Grindrod et al found that older patients, when introduced to new technologies, “struggled to think of a need for the applications in their own lives” [41].

When combined with our finding that patients considered data tracking to be “cumbersome”, this suggests that novel technologies will succeed only if they are highly intuitive, easy to learn, and unambiguously reduce the burden of work on the patient. Uploadable device data [42] or mining of personal data traces from phones and other technologies [43,44] may be effective ways of accomplishing this, especially given the fact that both patients and providers in our study recognized the additional perceived credibility of technologically measured data. The gamification trend in the health promotion and disease management literature is also potentially relevant [42,45,46]. Games that provide motivation to track learning opportunities, social support, or emotional coping support for dealing with data could potentially be useful for patients with chronic disease. However, designers of games for self-tracking may wish to consider our findings that patients often see data tracking as work and may perceive the data as having moral meaning that could be positive or negative. As noted by others, patients can have strong emotional responses to learning their own numbers and can feel judged by themselves and others [47-49]. Turning information tracking into a game might appear to trivialize important tasks, and “losing” in a game might amplify negative emotions. It might even be that some patients might prefer less emotionally charged technologies inspired by office or financial management software, which are explicitly designed to make necessary activities efficient and even pleasant while still treating those activities as work.

Our findings also have relevance for the literature on patients’ mental models of disease. As others [50,51] have pointed out, individuals work to make sense of their disease and health experiences, seeking a label or name, identifying its cause, establishing its probable timeline and consequences, and learning the extent to which it is manageable or curable. Over time, people use these insights to construct what have been called “common-sense models of disease” or “illness representations”, that is, explanations of health conditions that are internally coherent but that may or may not coincide with the biomedical model of the disease [50,51]. These illness representations can affect risk perceptions, coping behavior, management, and disease outcomes. Data tracking clearly offers the possibility of demonstrating the link between behavior and disease indicators (eg, between diet, medication administration, and blood glucose), thereby encouraging patients to develop a more biomedical model of their disease.

However, not all patients wanted to examine their data for this purpose. Our findings are striking in the degree to which medical data were shown to have extremely serious emotional implications for patients with MCC, sometimes serious enough to be associated with abandonment of data tracking altogether. “Bad” data values can be extremely upsetting, especially when those “bad” values have or are perceived to have some link to behavior. Patients’ language revealed the extent to which they use judgmental terms of sin and transgression to describe both their data and themselves. Furthermore, some of our patients noted with some surprise that their health care providers did not seem very interested in their self-logged data; others have noted that diabetes patients can interpret their providers’ preference for lab-measured hemoglobin A1c as meaning that self-monitoring was not important [48]. Peel et al found that counterintuitive blood glucose values confused patients and could lead to discontinuation of monitoring, as was reported by one of our patients [49].

One highly relevant study reports a trial of an electronic diabetes diary and information app, which incorporates some of the concepts we have recommended here [52]. In that trial, blood glucose measurements were automatically uploaded via Bluetooth from an electronic monitor, although food and exercise data had to be manually input. Counseling, including motivational interviewing, was added in one of the two technology arms. Nevertheless, after 4 months the app (with or without supplemental counseling) was not associated with changes in hemoglobin A1c levels [52]. The 18% attrition rate in this study may have resulted from the relatively heavy work burden of self-tracking the electronic data.

### Limitations

The sample was generally representative of the demographics of the MCC population. However, type 2 diabetes may have been more prevalent in our sample than in the national MCC population, in which type 2 diabetes occurs in three of the top nine pairs of chronic conditions and four of the top nine condition triads [53]. Interviews were also conducted in a US urban area and in English only, limiting the sample to patients comfortable in that language. These reasons may limit relevance to other populations, such as individuals in other countries with different health care systems, people in rural locations with different challenges in accessing health care, or people of other cultures or language groups.

### Conclusions and Implications

Developers of consumer health information technologies for data tracking (such as diet and exercise apps or blood glucose logs) often assume that a wide variety of patients will have unlimited enthusiasm for tracking their own health data via technology. However, adoption of new technologies does not always rapidly spread beyond computer-literate, highly motivated early adopters. We suggest that to make a public health impact, developers should be prepared to engage creatively with a variety of pragmatic and emotional issues to reach a broader audience that includes patients with chronic disease.

One recommendation is to explore ways to engage directly with the emotional impact associated with medical data, exploring ways not only to motivate progress but also cope with negative feelings. Developers should seek not to exacerbate negative feelings or judgments, look at creative ways to support positive feelings, and facilitate personal goal setting rather than imposing external goals. Technologies could integrate techniques such as motivational interviewing [54] that have been demonstrated to help patients establish personally relevant goals and action plans, rather than seeking to persuade patients to adopt their providers’ priorities. The behavioral economics literature can provide valuable guidance in leveraging effects such as framing, defaults, and behavioral “nudges” to promote engagement and better decision making [55].

Another suggestion is to provide different formats for different purposes. Patients who are building a conceptual understanding of disease might benefit from data-driven links with explanatory material or even simulations. Patients who are using data to check on goals might benefit from progress bars or visualized target thresholds. A relatively small number of patients (such as those adjusting insulin doses or high blood pressure medications [22]) will be using data for self-management; these individuals are most likely to be interested in reminders or alerts. Developing systems with the wrong purpose in mind appears likely to irritate patients rather than support them. For example, patients who have not established personally relevant goals are unlikely to welcome visualizations that depict their “progress”, and patients who are already well educated about their disease processes may prefer emotional and practical support to basic educational material.

It must still be recognized that older generations are not universally comfortable with electronic technologies and that many low-income patients still do not have access to them. For the foreseeable future, a significant subset of patients will lack access to information technology. This creates tremendous opportunities for exploring improved paper technologies. For example, scannable paper forms might ease the burden of tracking data on paper and be more widely used than mobile apps by some groups. Technologies that benefit only younger or more technologically sophisticated patients could have the potential to widen health disparities rather than narrow them. This issue of equity must be addressed in health information technology broadly, but especially in technology intended for personal health information tracking and management.

Finally, the concept of data tracking as yet another piece of patient “work” resonated strongly with the participants. Novel technologies are likely to be successful only if they clearly reduce inconveniences and burden for patients, helping them to accomplish their “work” more efficiently and effectively.

## Abbreviations

HIT: health information technology
HIV: human immunodeficiency virus
MCC: multiple chronic disease
